# Genetic and environmental contribution to phenotypic resemblance between Iranian couples: Tehran Cardiometabolic and Genetic Study (TCGS)

**DOI:** 10.1016/j.heliyon.2025.e42401

**Published:** 2025-02-01

**Authors:** Parisa Riahi, Amir Hossein Saeidian, Albert Tenesa, Carolyn T. Hogan, Michael March, Kamran Guity, Mahmoud Amiri Roudbar, Asieh Zahedi, Maryam Zarkesh, Farideh Neshati, Mehdi Hedayati, Fereidoun Azizi, Hakon Hakonarson, Maryam S. Daneshpour, Mahdi Akbarzadeh

**Affiliations:** a Cellular and Molecular Endocrine Research Center, Research Institute for Endocrine Molecular Biology, Research Institute for Endocrine Sciences, Shahid Beheshti University of Medical Sciences, Tehran, Iran; b Center for Applied Genomics (CAG), The Children's Hospital of Philadelphia, Philadelphia, PA, USA; c Department of Molecular and Human Genetics, Baylor College of Medicine., Houston, TX, USA; d The Roslin Institute, Royal (Dick) School of Veterinary Studies, The University of Edinburgh, Easter Bush Campus, Midlothian, Edinburgh, EH25 9RG, UK, Scotland, UK; e MRC HGU at the MRC IGMM, Western General Hospital, University of Edinburgh, Crewe Road South, Edinburgh, EH4 2XU, UK; f Division of Hepatology, Temple University Hospital, Philadelphia, PA, USA; g Center for Applied Genomics, The Children's Hospital of Philadelphia, Abramson Research Building, Suite 1016I, 3615 Civic Center Boulevard, Philadelphia, PA, 19104-4318, USA; h Department of Animal Science, Safiabad-Dezful Agricultural and Natural Resources Research and Education Center, Agricultural Research, Education & Extension Organization (AREEO), Dezful, Iran; i Endocrine Research Center, Research Institute for Endocrine Disorders, Research Institute for Endocrine Sciences, Shahid Beheshti University of Medical Sciences, Tehran, Iran; j Center for Applied Genomics (CAG), Children's Hospital of Philadelphia, 3615 Civic Center Blvd, Abramson Building, Philadelphia, PA, 19104, USA; k Department of Pediatrics, The Perelman School of Medicine, University of Pennsylvania, Philadelphia, PA, 19104, USA; l Division of Human Genetics, Children's Hospital of Philadelphia, Philadelphia, PA, 19104, USA; m Division of Pulmonary Medicine, Children's Hospital of Philadelphia, Philadelphia, PA, 19104, USA; n Faculty of Medicine, University of Iceland, Reykjavik, Iceland

**Keywords:** Assortative mating, Polygenic risk scores, Genetic AM, GWAS, Spousal resemblance, TCGS, Cohabitation effect, Height

## Abstract

**Objective:**

To provide an applied framework for assessing the genetic contribution to assortative mating (AM) using height as a model trait and disclose the trace of certain pieces of evidence of AM in the form of the shared environmental effects from long-term cohabitation on spouses’ anthropometric traits and lipid serum levels.

**Methods:**

2315 genotyped couples were extracted from the Tehran Cardiometabolic Genetic Study (TCGS). Pearson correlation analysis was used to assess the relationship between spouses' height. The GCTA-GREML was used to assess the SNP-based heritability of individual and spousal heights with AM adjustments. We used a recent GWAS meta-analysis of ∼5.4M individuals of height to calculate polygenic risk scores (PRS) for spouses’ height. A subset of 1038 spouses out of 2315 couples were subsequently selected to enter the longitudinal resemblance, to be assessed in terms of their anthropometric traits and lipid serum levels in a 15-year follow-up. We conducted a Bayesian hierarchical meta-analysis for each time point to assess the validity of the increasing trend of the longitudinal association.

**Results:**

The correlation coefficient of height between spouses was estimated as r = 0.248. We found that a person's genotype determines 6.15 % of the variation in the spouse's height. Furthermore, correlation between PRS of individuals showed a statistical association between an individual’s genotype and their spouse’s genotype (R2 = 4 %) across 1,982 couples with only one genotyped spouse, achieving approximately half of the theoretical maximum accuracy. Long-term spousal resemblance revealed an increasing trend for correlation between husbands and wives in terms of their lipid serum level and obesity-related traits.

**Conclusion:**

Our findings support the AM hypothesis for height with a significant spousal correlation and show that selecting the spouse's height is genetically determined. Besides, we provide data showing that AM is predicted to result in a10 % increase in the heritability of height, which is related to the assortative nature of alleles in the population and not to the segregation of genetic variations. Finally, as one of the evolutionary consequences of AM, long-term spousal resemblance provided an increasing trend for correlation between spouses in terms of their lipid serum level and obesity-related traits.

## 1 Introduction

Assortative mating (AM) is a phenomenon in which individuals select mates with similar phenotypes to their own. This includes physical traits (phenotypic assortment), meaning partner choice is based on observable characteristics, not just physical but also non-physical traits. Additionally, people may choose partners who share socio-cultural or socio-economic backgrounds. This is known as social homogamy, where partners are selected because they share similar social or environmental backgrounds, such as living in the same communities. AM is recognized as the most common deviation from random mating in Western societies [[1], [2], [3], [4]]. AM is most often positive, occurs on various physical and non-physical traits, strengthens the mating bond, and increases fertility [5]. A positive AM with shared environmental conditions (i.e., cohabitation effects), age effects between spouses with specific traits and backgrounds, or a combination of these factors may account for the fact that spouses tend to be more similar over time than one would expect if spouses were chosen randomly from a large population [6]. Positive AM increases genetic and phenotypic variance in a population by allowing individuals with similar traits to pair. This results in offspring inheriting more extreme traits, causing genetic variance. AM does not introduce new variants but redistributes existing variations, causing a shift in the population's genetic structure. Consistent AM can influence allele frequencies and alter trait distributions [[7], [8], [9]].

Among physical traits, height has consistently been shown to be a strong determinant of AM, with studies reporting significant correlations in spousal height across various populations [10,11]. Height is a highly heritable and polygenic trait, making it an ideal model for understanding the interplay between genetic and environmental factors in AM. According to a published report involving 13,068 adult couples living together, a person's genotype is associated with their spouse's height, with both genetic and environmental factors contributing to the height association identified between spouses [12].

Since AM can result from various mechanisms, including phenotypes based on mate choice or social homogamy, where individuals mate based on social or environmental origins, the causes and genetic consequences of AM remain to be addressed [[13], [14], [15], [16]]. Further, as spousal resemblance could arise from AM, shared environmental influences, or the different survival of marriages of spouses that are more similar to one another, longitudinal cohorts may better provide the AM influences on observable traits of couples during long periods [6,17].

Here, we aimed to investigate the genetic contributions to AM using height as a model trait. We hypothesized that both genetic factors contribute significantly to one's behavior in choosing a mate. Specifically, we aimed to quantify the genetic contribution using SNP-based heritability and polygenic risk scores (PRS) and assess the environmental contribution through longitudinal spousal resemblance in anthropometric traits and lipid serum levels.

## 2 Results

In the phenotypic correlation of spouses, the correlation between one individual's height and their spouse's height is estimated to be 0.248 (CI:0.209–0.286, P-value <0.001). This means that about R^2^ = 6.15 % of variations in an individual's height can be explained by their spouse's height. Fig. 3 compares phenotypic and genotypic evidence for height-related AM between the TCGS and UK Biobank populations. The phenotypic correlation between spouses for height (R-squared) is slightly lower in the TCGS cohort compared to the UK Biobank.

The SNP-based heritability of height by univariate linear models for the 4630 individuals (2315 couples) available using the 631,579 autosomal genetic markers that passed the QC protocol is 0.24 (SE: 0.044, P-value <0.001), meaning that about 24 % of variations in one's height can be estimated using their genotypes. This estimate is approximately half of the SNP-based heritability observed in the UK Biobank cohort. However, the SNP-based heritability of the spouse's height is estimated to be 0.08 (SE: 0.005, P-value <0.001), which exceeds that of the UK Biobank. These differences highlight potential variations in environmental influences and cultural norms affecting AM between the two populations. Our estimate reflects the genetic similarity between spouses that arises as a consequence of AM. Rather than indicating individual-level mate selection behavior directly influenced by genotype, this value demonstrates the non-random pairing of individuals with genetically similar traits, such as height, within the population. Fig. 4 illustrates the SNP-based heritability estimates for height under two conditions: AM and random mating. These values demonstrate how AM contributes to increased heritability (10 %) by changing the genetic architecture of the population.

This increase reflects the assortative nature of alleles in the population rather than the segregation of genetic variations. Approximately 22 % of the heritability for height in Iran can be attributed to the non-random assortment of alleles caused by AM.

Next, we used the most recent summary statistics of the GWAS meta-analysis (∼5.4M individuals, 1,377,307 SNPs) to compute polygenic risk scores of heights for couples with only one genotyped spouse.

The correlation between the PRS of the individual and their spouse's height (1892 couples) is estimated to be 0.2057 (CI: 0.1597–0.2507, P-value <0.001). This indicates that approximately 4 % of the variation in an individual's height is statistically associated with their spouse's genotype. To put it simply, this result reflects a statistical association arising from AM patterns rather than a direct genetic effect of one partner on the other's height. Consequently, this finding does not imply causation but highlights the genetic alignment between spouses that is characteristic of AM. Furthermore, this estimate represents about 50 % of the theoretical maximum accuracy of SNP-based heritability.

**Spousal resemblance for a 15-year follow-up:** Results for spousal resemblance for the 15-year follow-up (5 measurement time-points) are presented in Fig. 5(a) and (b). Fig. 5 (a) shows the longitudinal spousal resemblance in terms of adiposity-related traits, including waist circumference (WC), body mass index (BMI), and waist-to-hip ratio (WHR), measured at five-time points over the 15-year follow-up. And, Fig. 5 (b) illustrates spousal resemblance for lipid serum levels, including triglycerides (TG), total cholesterol (TC), high-density lipoprotein (HDL), and low-density lipoprotein (LDL). Together, these findings show an increasing trend for correlations over time. However, these correlations mostly do not differ significantly for two successive time points, but they do differ significantly after at least two time points. Although nominally significant, we checked the significance of our effect sizes after correction for multiple tests based on the Bonferroni correction method. Given that we had analyzed seven traits, the Bonferroni-corrected alpha threshold for each time point was set at corrected-alpha = 0.05/7 = 0.00714. After Bonferroni correction, the effect sizes are still significant for almost all of the tests. Detailed results of multiple tests are provided in the Supplementary File 1.

Results for the Bayesian hierarchical meta-analysis are provided in Table 1, which validates the increasing trend of Pearson correlation coefficients for longitudinal spousal resemblance regarding the anthropometric traits and lipid serum levels. Forest and funnel plots of the corresponding meta-analyses are provided in Supplementary File 2.

**Table 1:** The results of Bayesian hierarchical meta-analysis.

Time point	Number of traits	Pooled r∗∗ (Posterior median of r)	Heterogeneity test *τ* (*C·I 95 %*)	Bayesian sensitivity analysis
**First**	7	0.085	0.605∗	Robust
**Second**	7	0.089	0.729∗	Robust
**Third**	7	0.103	0.594∗	Robust
**Fourth**	7	0.144	0.584∗	Robust
**Fifth**	7	0.148	0.623∗	Robust

∗Significant level is considered < 0.05.

∗∗r: Pearson Correlation Coefficient.

## 3 Discussion

The present study pioneered the examination of the genomic contribution of AM among TCGS spouses in terms of their height and provided evidence for a long-term spousal resemblance regarding their anthropometric traits and lipid serum levels. Our results support the AM hypothesis for height, as there was a significant correlation between individuals and their spouses’ height. Here, we provide data that illustrate the extent to which the selection of mate height is influenced by genetic factors, as evidenced by the phenotypic assortment based on height likeness. The genetic associations observed between spouses are a result of this phenotypic assortment.

Numerous studies have reported positive correlations among couples for height and have indicated that height matters when evaluating the attractiveness of potential spouses, suggesting that mate height is likely to play a significant role [11,[18], [19], [20], [21], [22], [23], [24], [25], [26], [27], [28], [29], [30], [31], [32], [33], [34], [35], [36], [37], [38], [39], [40], [41], [42], [43], [44], [45], [46], [47], [48], [49], [50], [51], [52], [53], [54], [55], [56]]. A recent meta-analysis stated that AM for height in Western populations indicates a slightly higher degree (r = 0.25) than in non-Western populations (r = 0.21) [11]. Our findings demonstrate a significant positive correlation in height between husbands and wives, supporting the presence of positive AM for height in the Iranian population (r = 0.248). The association implies that height-based partner selection is not limited by geography or culture, as it closely matches the reported averages from Western people. Height is a universally desired attribute in mate selection, as evidenced by the similarity between the Iranian and Western correlation values. However, the meta-analysis's somewhat lower correlations for non-Western groups might be due to variations in social behaviors, cultural norms, or the role of family preferences in arranged marriages, all of which are more common in non-Western cultures.

Several studies have tried to detect AM on a molecular genetic level by estimating spousal resemblance based on SNP information [12,[55], [56], [57]]. These studies report that spouses exhibit greater genetic similarity on genome-wide SNPs than expected under random mating. Our findings show that SNP-based heritability for the height of individuals' spouses is estimated as 0.08, which reflects upon the degree of attraction to mates of similar height by one's genotypes.

Comparing our results with the most recent reports on the phenotypic correlation of height, height and spouse's height SNP-based heritability from UK Biobank [12,58], we realized that SNP-based heritability of height for TCGS participants is almost half of that of UK Biobank subjects. As there are no significant differences between the number of SNPs in models, we can say this might not be a contributor. Although the sample size of TCGS was proportionately smaller than that of UK Biobank, this should have affected the standard errors, which did not. Hence, to check the reliability of our results and also check the source of this significant difference, we measured the SNP-based heritability of height, which is calculated under AM, with the one that is calculated independently (under random mating).

To do so, we estimated the pedigree-based heritability of height for Iranian subjects (∼14k), which was 0.45 and consistent with our findings for SNP-based heritability. Using pedigree-based heritability, we estimated independent heritability and compared our results with those of UK Biobank. These measures satisfied our results for SNP-based heritability in TCGS spouses. Considering these facts, there may be more environmental variance in TCGS than in UK Biobank. In contrast to the more homogeneous and generally higher socioeconomic class of UK Biobank participants, the TCGS cohort exhibits greater environmental variability, including a wider range of socioeconomic statuses, educational levels, and cultural behaviors. Hence, in comparison to the UK Biobank, TCGS has lower SNP-based heritability estimates for height due to the higher environmental variance caused by this environmental diversity [[59], [60], [61], [62]].

To strengthen our results regarding the genetic contribution of AM, we fitted a prediction model. We believed that if some parts of variations in height can be explained by one's spouse's genotype, then to some extent, we would be able to predict one's height using their spouse's genotype. To do so, we used the most recent summary statistics of the GWAS meta-analysis (∼5.4M individuals, 1,377,307 SNPs) to compute polygenic risk scores of heights for couples with only one genotyped spouse.

Positive AM can shape the direction and intensity of natural selection on traits by amplifying their variance within populations. When combined with shared environmental influences, it can deepen the long-term resemblance between spouses, particularly for traits tied to specific backgrounds and lifestyles [63,64].

Recent studies have revealed the long-term cohabitation effect between spouses living in each other's immediate environment in terms of body obesity, smoking, cardiovascular disease, and its risk factors such as lipid serum levels, blood glucose, blood pressure, etc. [[65], [66], [67], [68], [69], [70]]. Similarities between spouses may result from a similar environment, shared behaviors, and even AM. If this concordance was primarily a result of cohabitation, then it should grow as the time spent by couples increases. Although the degree of consanguinity between spouses varies between nations, spouses are often genetically unrelated yet share the same environment.

Convergence refers to the mechanism by which phenotypic resemblance between spouses may increase over time, often attributed to shared environmental factors and behavioral synchronization during cohabitation [17,65].

In our study, the spousal resemblance results for the five measurement time-points in the 15-year follow-up demonstrate an increasing tendency for correlations with time. Our results reveal that spousal resemblance is influenced by both behavioral synchronization and environmental homogeneity among spouses. Prolonged cohabitation often leads to similar metabolic profiles and lipid serum levels, with health-related discussions and shared interventions strengthening these correlations. The non-random pairing due to AM at the time of mate selection sets the foundation for these observed trends. Genetic predispositions interact with environmental exposures, particularly in traits like lipid levels and adiposity. The strength of this effect may vary depending on the trait's nature, highlighting the importance of considering both genetic and environmental components in interpreting spousal resemblance. We emphasize that while convergence may contribute to the spousal resemblance in dynamic traits, such as lipid levels and obesity-related metrics observed in our longitudinal analysis, it does not have any role in static traits like height.

By examining AM in an Iranian population, our study fills this important knowledge gap and offers fresh perspectives on the genetic and phenotypic aspects of AM in Iran as a Middle Eastern country. We provide important evidence on spousal resemblance for adiposity-related traits and lipid serum levels by utilizing data from the Tehran Cardiometabolic Genetic Study (TCGS), one of the biggest and most extensive longitudinal cohorts in Iran, which expands the understanding of AM globally beyond primarily Western frameworks [59].

Although our study has successfully fulfilled its objectives, it has certain limitations compared to other studies. Our sample size (n = 4630) for fitting the bivariate linear mixed model was approximately small, and our sampling variance was large in both GCTA(71) and LDSC (Linkage Disequilibrium Score Regression) [72]. Hence, we were not able to provide the shared genetic determinants between the height of participating individuals and their respective spouse's height. Also, we could not capture a large sample of spouses for our follow-up to monitor their obesity-related traits and lipid serum levels, as our cohort, as a longitudinal study, is subject to some missingness. Regarding spousal resemblance, we did not apply a formal correction for multiple testing, and we considered each phenotype independently due to its distinct biological implications.

Future research should expand beyond the Iranian population to non-Western and underrepresented regions to understand the universality and variability of AM patterns. Longitudinal data collection for a wider range of traits, such as psychological characteristics and lifestyle behaviors, could help disentangle the complex interplay between genetic and environmental influences on spousal resemblance. Understanding environmental factors like urbanization, dietary habits, and healthcare access could have implications for public health initiatives. Advanced genetic tools could enable a more granular exploration of AM's genetic architecture and evolutionary consequences.

## 4 Conclusion

Our study provides a statistically significant association between individuals’ height and that of their spouses, which validates the AM hypothesis. We also demonstrate that selecting a spouse with a specific height is at least in part genetically determined. As a result of AM, long-term spousal resemblance (∼15 years), we show an increasing trend of correlation between husbands and wives in lipid serum level and obesity-related traits.

## 5 Methods and materials

**Study subjects:** Data were selected from the Tehran Cardiometabolic and Genetic Study (TCGS), an ongoing cohort study that is running in the framework of the Tehran Lipid and Glucose Study (TLGS) [59,[73], [74], [75]]. In the TLGS, participants from District 13 of Tehran, Iran, have been followed for important cardiovascular and metabolic health events, such as obesity and dyslipidemia, throughout the past 23 years. Six follow-up periods were conducted, with a roughly three-year space between two consecutive phases with 20276 participants (started in 1999). The study's full scope and methodology are described elsewhere [76].

**Fig. 1:**
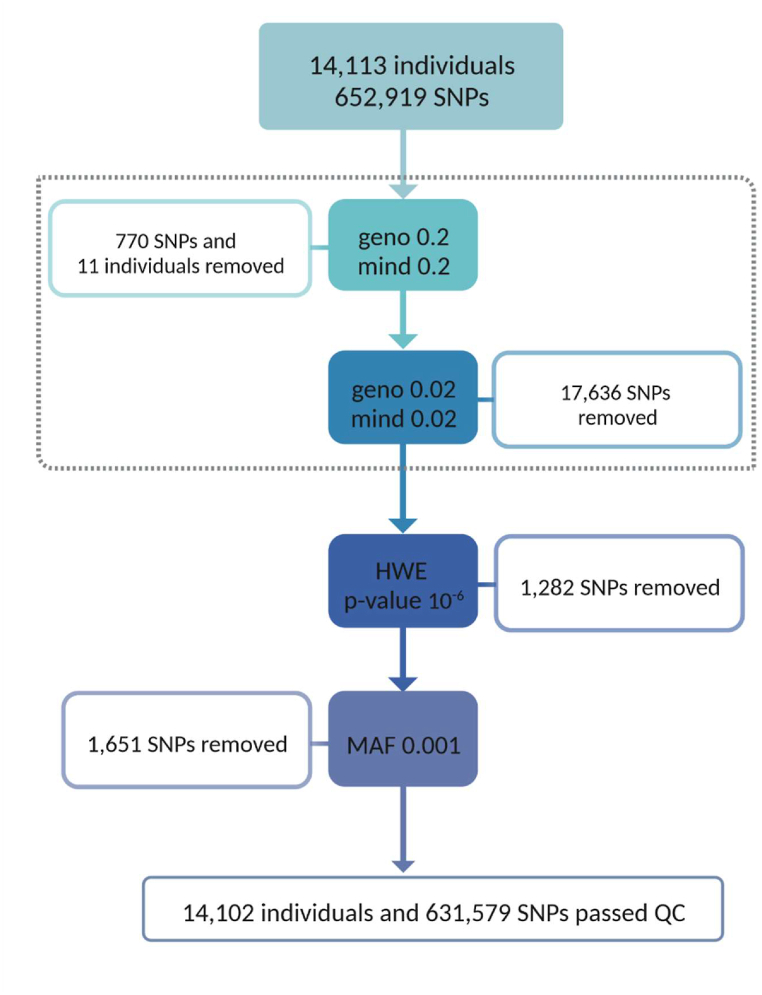
Quality control process of TCGS participants.

**Genotyping and QC procedure:** 14,113 individuals were genotyped at the deCODE genetic company using HumanOmniExpress-24-v1 BeadChips to genotype blood samples, which has provided us with 652,919 single nucleotide polymorphisms given an average distance of 4 kilobases between markers. The Plink software was used for the quality control of the individuals and variants, providing us with 14,102 individuals with 631,579 markers [77]. QC procedure is presented in Fig. 1.

**Phenotype definition:** The height of individuals was measured according to TLGS protocols [76]. For checking spousal resemblance, we have included waist circumference (WC) (cm), and body mass index (BMI): $\frac{weight\left(kg\right)}{{height\left(m\right)}^{2}}$ ,waist-to-hip ratio (WHR): $\frac{WC\;\left(cm\right)}{hip\;circumference\left(cm\right)}$, total cholesterol (TC) which was assessed using an enzymatic colorimetric method assessing cholesterol esterase and oxidase, triglyceride (TG) for which glycerol phosphate oxidase was utilized, high-density lipoprotein cholesterol (HDL-C) which was determined after apolipoprotein B-containing lipoproteins were precipitated with phosphotungstic acid, and low-density lipoprotein cholesterol (LDL-C) which was calculated using a modified Friedewald formula in conjunction with TG, TC, and HDL-C [74,76].

**Spouses’ selection and 15-year follow-up:** We selected 6126 couples from nuclear families of the Tehran Cardiometabolic Genetic Study (TCGS), where each family included parents and at least one child. The study involved two main stages [1]: Assortative Mating (AM) analysis, which included (1.1) correlations and (1.2) prediction, and [2] Spousal Resemblance analysis.

**Fig. 2:**
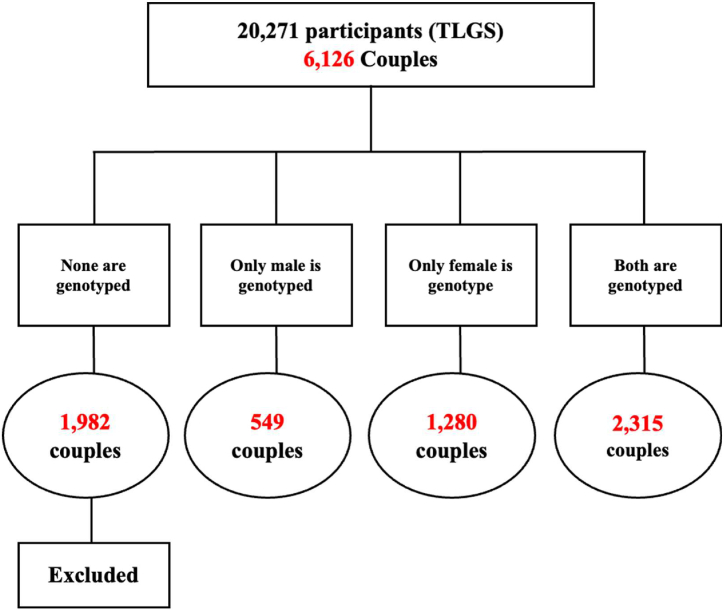
Study participants' selection flowchart.

Out of the initial 6126 couples, 1982 were excluded from further analysis as none of the individuals in these couples were genotyped, leaving us with 4144 couples with at least one genotyped individual. Specifically, 1829 couples had at least one genotyped spouse (1280 couples had only females genotyped, and 549 couples had only males genotyped). These 1829 couples entered the prediction stage of the study. Additionally, 2315 couples, where both spouses were genotyped, entered the first stage of our study (Fig. 2).

**Fig. 3:**
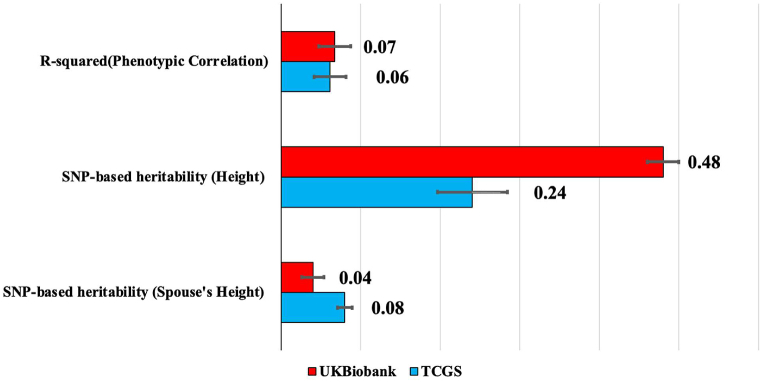
Comparison of phenotypic and genotypic evidence for height-related assortative mating between Iranians and individuals from the UK Biobank.

**Fig. 4:**
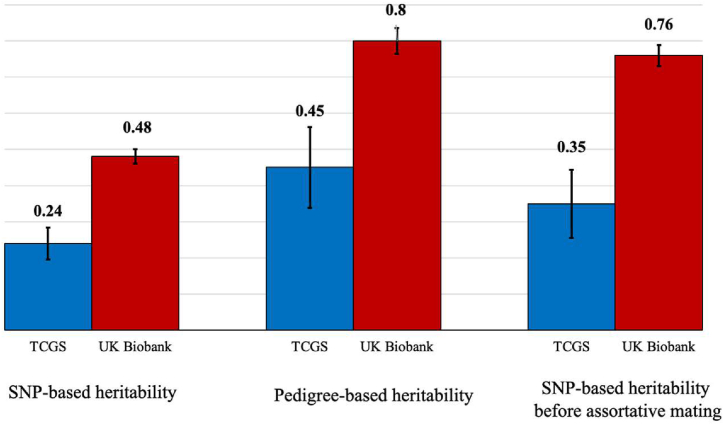
Reliability of the evidence for height-related assortative mating in Iranian couples.

**Fig. 5 (a):**
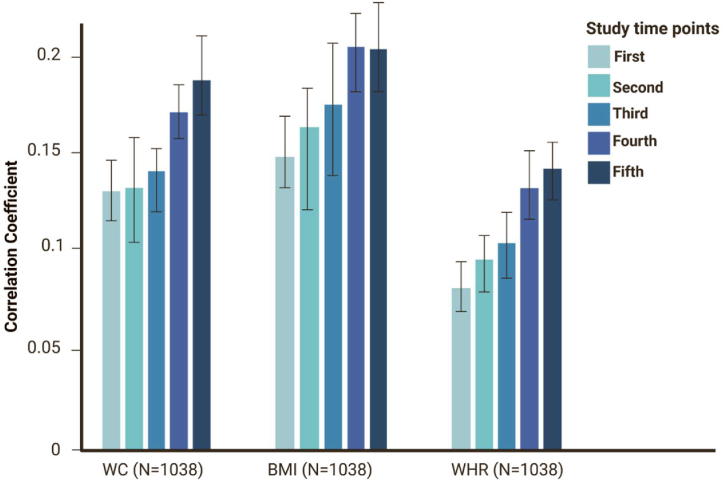
Spousal resemblance in terms of adiposity-related traits at five time points of follow-up (WC: Waist Circumference, BMI: Body Mass Index, WHR: Waist-to-Hip Ratio).

**Fig. 5 (b):**
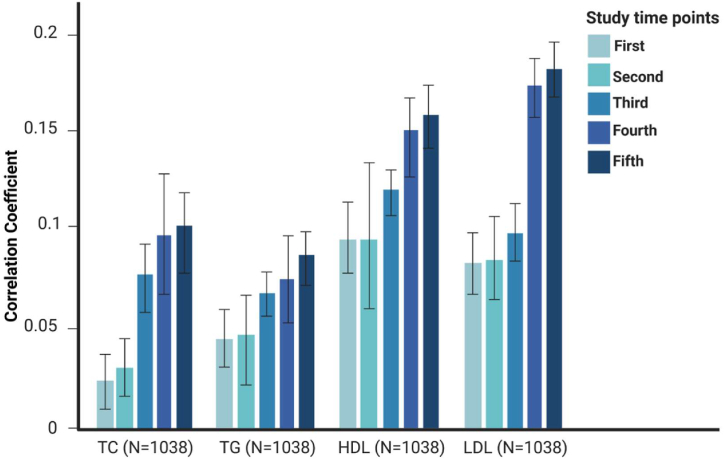
Spousal resemblance in terms of lipid serum levels at 5 time points of follow-up (TG: Triglyceride, TC: Total Cholesterol, HDL: High-density Lipoprotein, LDL: Low-density Lipoprotein).

In the second stage, we selected 1038 nonconsanguineous couples (unrelated spouses) from the 2315 genotyped couples to enter the longitudinal resemblance phase of the study. The identification of nonconsanguineous couples was based on detailed family history data provided by the participants. This selection was crucial to avoid confounding effects from shared family genetics and to ensure the validity of our genetic analyses. Both spouses in these selected couples participated in a 15-year follow-up, where their anthropometric traits and lipid serum levels were assessed at five-time points with 3-year intervals. This follow-up period started in 2002 (2nd phase of TLGS) and ended in 2017 (6th phase of TLGS).

### 5.1 Statistical analysis

**Correlation analysis:** Pearson correlation coefficient, along with confidence intervals, was reported to assess the relationship between height, WC, BMI, WHR, TC, TG, HDL-C, and LDL-C in males and females. The statistically significant level was considered 0.05 for each individual test.

Multiple testing correction: We applied the Bonferroni correction method to account for the potential inflation of Type I error due to multiple comparisons. This approach adjusts the significance threshold to control the **family-wise error rate (FWER)**. Specifically, the threshold for significance was set at α/m, where α is the desired significance level (0.05) and m is the total number of comparisons performed in the analysis. This ensures that the overall probability of obtaining false positives is minimized.

We applied this correction to the statistical tests assessing spousal resemblance for adiposity traits (BMI, WC, WHR) and lipid serum levels (TG, HDL-C, LDL-C, TC) across five time points.

**Univariate linear mixed model**: To estimate the heritability of height, we used the GCTA-GREML (Genome-wide Complex Trait Analysis - Genomic-relatedness-based restricted maximum likelihood) method. This involved estimating the genomic relationship matrix (GRM) from 2315 spouses using 631,579 genetic markers, following stringent quality control procedures [71].

Our model included several fixed effects such as sex, age, and the first 10 principal components (PCs) to account for population structure. The SNP markers were represented as M = {m_ij_}, where i = 1, …,n denotes individuals and j = 1, …, pg denotes the number of genetic markers.

The genetic contribution was modeled using a Gaussian random-effect model with a specific covariance structure. The genomic random effect was included in the model, representing a linear regression on the SNP markers. This was specified as $g=N\left(0,G\sigma_{g}^{2}\right)$ and the residuals as $e∼N\left(0,{I\sigma }_{e}^{2}\right)$, where GG is the genomic relationship matrix, I is an identity matrix, and $\sigma_{g}^{2}$ and $\sigma_{e}^{2}$ are the genetic and residual variances, respectively. The response variable y = {y_i_} was defined as the height of the ith individual [78].

**Polygenic risk score (PRS):** To estimate the PRS of the spouses with only one genotyped spouse, we used the summary statistics of a recent GWAS meta-analysis (∼5.4M individuals, 1,377,307 SNPs) on height and extracted the effect size of ∼137k SNPs, to compute polygenic risk scores of heights for both males and females using PLINK software [79,80]. Both base (summary statistics from a large-scale GWAS meta-analysis) and target (genotyped individuals from the TCGS cohort) data were QCed before entering the PRS calculation phase, the steps of which are described in Ref. [81].

Estimation of heritability adjusted for AM: The heritability of height, before the population started AM and reached an equilibrium ($h_{0}^{2}$), was estimated as $h_{0}^{2}=h^{2}\left[\frac{1-m}{1+mh^{2}}\right]$, where h^2^ is the SNP-based heritability of height, and m is the correlation of genetic values among spouses [12,15].

**Bayesian hierarchical meta-analysis:** To assess the validity of the increasing trend of the longitudinal association between husbands and wives for their adiposity-related traits and lipid serum levels, we conducted a Bayesian hierarchical meta-analysis for each time-point. When potential heterogeneity among traits was present, Bayesian hierarchical meta-analysis effectively accommodated and modeled this variability. A more robust framework for analyzing data with such characteristics is provided by the Bayesian approach, in contrast to the classic random effects method, which can occasionally struggle with the degree of heterogeneity present in our traits. Furthermore, we justified this approach based on the relatively small number of traits analyzed.

To model the between-variable variability, Pearson correlation coefficients as the effect size of the association with a 95 % credible interval have been considered following a normal distribution. The pooled results of Bayesian hierarchical meta-analysis were obtained as the median of the posterior distribution of Pearson correlation coefficients. The heterogeneity between the traits was identified with Cochran's Q and I^2^ tests. Also, the robustness of Bayesian analysis was confirmed by sensitivity analysis. Bayesian meta-analysis was conducted using the “*bayesmeta*” R package [82].

## CRediT authorship contribution statement

**Parisa Riahi:** Writing – review & editing, Writing – original draft, Software, Methodology, Formal analysis, Conceptualization. **Amir Hossein Saeidian:** Software, Formal analysis, Conceptualization. **Albert Tenesa:** Investigation. **Carolyn T. Hogan:** Writing – original draft, Formal analysis, Conceptualization. **Michael March:** Investigation. **Kamran Guity:** Investigation, Data curation. **Mahmoud Amiri Roudbar:** Investigation. **Asieh Zahedi:** Data curation. **Maryam Zarkesh:** Writing – review & editing, Validation. **Farideh Neshati:** Writing – review & editing, Validation. **Mehdi Hedayati:** Writing – review & editing, Validation. **Fereidoun Azizi:** Writing – review & editing, Validation. **Hakon Hakonarson:** Writing – review & editing, Supervision, Methodology. **Maryam S. Daneshpour:** Writing – review & editing, Conceptualization. **Mahdi Akbarzadeh:** Writing – review & editing, Writing – original draft, Software, Project administration, Formal analysis, Conceptualization.

## Availability of data and materials

The datasets generated and/or analysed during the current study are not publicly available due to the privacy of research participants but are available from the corresponding author at reasonable request.

**Table:** Abbreviations

**Abbreviation**	**Full Version**
**AM**	Assortative Mating
**BMI**	Body Mass Index
**CI**	Confidence Interval
**GRM**	Genomic Relatedness Matrix
**GWAS**	Genome-Wide Association Study
**HDL**	High-Density Lipoprotein
**LDL**	Low-Density Lipoprotein
**PC**	PrincipalComponent
**PRS**	Polygenic Risk Score
**LDSC, or LDSR**	Linkage Disequilibrium Score Regression
**QC**	Quality Control
**SD**	Standard Deviation
**SE**	Standard Error
**TC**	Total Cholesterol
**TCGS**	Tehran Cardiometabolic and Genetic Study
**TG**	Triglyceride
**TLGS**	Tehran Lipid and Glucose Study
**WC**	Waist Circumference
**WHR**	Waist-to-Hip Ratio

## Consent for publication

Not applicable.

## Funding

Not applicable.

### Ethics declarations

Ethics approval and consent to participate:

The local ethics committee approved this study at the Research Institute for Endocrine Sciences, Shahid Beheshti University of Medical Sciences (Research Approval Code: 28778 & Research Ethical Code: IR.SBMU.ENDOCRINE.REC.1400.083). In this study, all participants provided written informed consent to participate. The research has been performed in accordance with the Declaration of Helsinki.

## Declaration of competing interest

The authors declare the following financial interests/personal relationships which may be considered as potential competing interests: Mahdi Akbarzadeh reports statistical analysis was provided by Shahid Beheshti University of Medical Sciences Research Institute for Endocrine Sciences. Mahdi Akbarzadeh reports a relationship with Shahid Beheshti University of Medical Sciences Research Institute for Endocrine Sciences that includes: board membership. If there are other authors, they declare that they have no known competing financial interests or personal relationships that could have appeared to influence the work reported in this paper.

## Footnotes

Appendix A Supplementary data to this article can be found online at https://doi.org/10.1016/j.heliyon.2025.e42401.
