# Supplementary material for: Genetic and environmental contribution to phenotypic resemblance between Iranian couples: Tehran Cardiometabolic and Genetic Study (TCGS)
Source: Heliyon. 2025 Feb 1;11(3):e42401. doi: 10.1016/j.heliyon.2025.e42401 (PMC11849086; doi:10.1016/j.heliyon.2025.e42401)

**Genetic and Environmental Contribution to Phenotypic Resemblance between Iranian Couples: Tehran Cardiometabolic and Genetic Study (TCGS)**

**Running Title:** Height-related Assortative Mating and its Implications

1. **Parisa Riahi,** MSc, Cellular and Molecular Endocrine Research Center, Research Institute for Endocrine Molecular Biology, Research Institute for Endocrine Sciences, Shahid Beheshti University of Medical Sciences, Tehran, Iran. Email: [parisaariyahii@gmail.com](mailto:parisaariyahii@gmail.com)
2. **Amir Hossein Saeidian,** ^✝^, Ph.D., E-mail: saeidiana@chop.edu

- Center for Applied Genomics (CAG), The Children’s Hospital of Philadelphia, Philadelphia, PA, USA
- Department of Molecular and Human Genetics, Baylor College of Medicine., Houston, TX

1. **Albert Tenesa,** Ph.D, The Roslin Institute, Royal (Dick) School of Veterinary Studies, The University of Edinburgh, Easter Bush Campus, Midlothian, Edinburgh, EH25 9RG, Scotland. MRC HGU at the MRC IGMM, Western General Hospital, University of Edinburgh, Crewe Road South, Edinburgh, EH4 2XU, UK. [albert.tenesa@ed.ac.uk](mailto:albert.tenesa@ed.ac.uk)
2. **Carolyn T. Hogan,** MD, Division of Hepatology, Temple University Hospital, Philadelphia, PA, USA. Email: [carolyn.hogan@tuhs.temple.edu](mailto:carolyn.hogan@tuhs.temple.edu)
3. **Michael March,** Center for Applied Genomics, The Children’s Hospital of Philadelphia, Abramson Research Building, Suite 1016I, 3615 Civic Center Boulevard, Philadelphia, PA, 19104-4318, USA, Email: marchm@chop.edu
4. **Kamran Guity,** MSc, Cellular and Molecular Endocrine Research Center, Research Institute for Endocrine Molecular Biology, Research Institute for Endocrine Sciences, Shahid Beheshti University of Medical Sciences, Tehran, Iran. Kamran.Guity@gmail.com
5. **Mahmoud Amiri Roudbar,** Ph.D, Department of Animal Science, Safiabad-Dezful Agricultural and Natural Resources Research and Education Center, Agricultural Research, Education & Extension Organization (AREEO), Dezful, Iran, Email: [mahmood.amiri225@gmail.com](mailto:mahmood.amiri225@gmail.com)
6. **Asieh Zahedi,** MSc, Cellular and Molecular Endocrine Research Center, Research Institute for Endocrine Molecular Biology, Research Institute for Endocrine Sciences, Shahid Beheshti University of Medical Sciences, Tehran, Iran. Email: asiyezahedi18@gmail.com
7. **Maryam Zarkesh,** Ph.D., Cellular and Molecular Endocrine Research Center, Research Institute for Endocrine Molecular Biology, Research Institute for Endocrine Sciences, Shahid Beheshti University of Medical Sciences, Tehran, Iran. Email: ​[zarkesh@endocrine.ac.ir](mailto:zarkesh@endocrine.ac.ir)
8. **Farideh Neshati,** MSc, Cellular and Molecular Endocrine Research Center, Research Institute for Endocrine Molecular Biology, Research Institute for Endocrine Sciences, Shahid Beheshti University of Medical Sciences, Tehran, Iran. Email: faride.neshati@gmail.com
9. **Mehdi Hedayati,** Ph.D., Cellular and Molecular Endocrine Research Center, Research Institute for Endocrine Molecular Biology, Research Institute for Endocrine Sciences, Shahid Beheshti University of Medical Sciences, Tehran, Iran. Email: ​ hedayati47@gmail.com
10. **Fereidoun Azizi,** MD, Endocrine Research Center, Research Institute for Endocrine Disorders, Research Institute for Endocrine Sciences, Shahid Beheshti University of Medical Sciences, Tehran, Iran. Email: azizi@endocrine.ac.ir
11. **Hakon Hakonarson,** M.D, Ph.D., E-mail: [hakonarson@chop.edu](mailto:hakonarson@chop.edu)

- Center for Applied Genomics (CAG), Children’s Hospital of Philadelphia, 3615 Civic Center Blvd, Abramson Building, Philadelphia, PA, 19104, USA
- Department of Pediatrics, The Perelman School of Medicine, University of Pennsylvania, Philadelphia, PA, 19104, USA
- Division of Human Genetics, Children’s Hospital of Philadelphia, Philadelphia, PA, 19104, USA
- Division of Pulmonary Medicine, Children’s Hospital of Philadelphia, Philadelphia, PA, 19104, USA
- Faculty of Medicine, University of Iceland, Reykjavik, Iceland

1. **Maryam S Daneshpour*,** Ph.D., Cellular and Molecular Endocrine Research Center, Research Institute for Endocrine Molecular Biology, Research Institute for Endocrine Sciences, Shahid Beheshti University of Medical Sciences, Tehran, Iran. Email: [daneshpour@sbmu.ac.ir](mailto:daneshpour@sbmu.ac.ir)
2. **Mahdi Akbarzadeh*,** Ph.D., Cellular and Molecular Endocrine Research Center, Research Institute for Endocrine Molecular Biology, Research Institute for Endocrine Sciences, Shahid Beheshti University of Medical Sciences, Tehran, Iran. Email: ​ [akbarzadeh.ms@gmail.com](mailto:akbarzadeh.ms@gmail.com)

*Corresponding authors:

- First: Mahdi Akbarzadeh*, Ph.D., Cellular and Molecular Endocrine Research Center, Research Institute for Endocrine Molecular Biology, Research Institute for Endocrine Sciences, Shahid Beheshti University of Medical Sciences, Tehran, Iran. Email: ​[akbarzadeh.ms@gmail.com](mailto:akbarzadeh.ms@gmail.com), P.O. Box: 19395-4763, 1985717413, Tel: +98 (21) 22432500, Fax: +98 (21) 22402463
- Second: Maryam S Daneshpour, Ph.D., Cellular and Molecular Endocrine Research Center, Research Institute for Endocrine Molecular Biology, Research Institute for Endocrine Sciences, Shahid Beheshti University of Medical Sciences, Tehran, Iran. Email: [daneshpour@sbmu.ac.ir](mailto:daneshpour@sbmu.ac.ir)

**Bayesian Hierarchical Meta-Analysis results**

**(*Longitudinal Spousal Resemblance in Anthropometric Traits and Lipid Serum Levels*)**


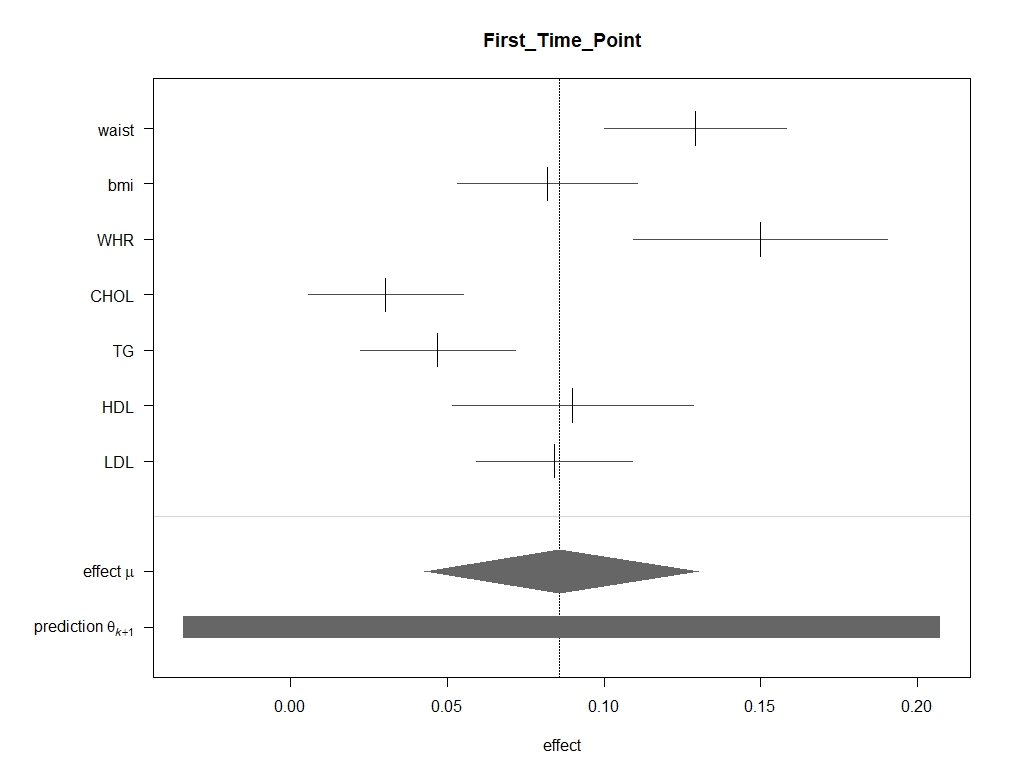


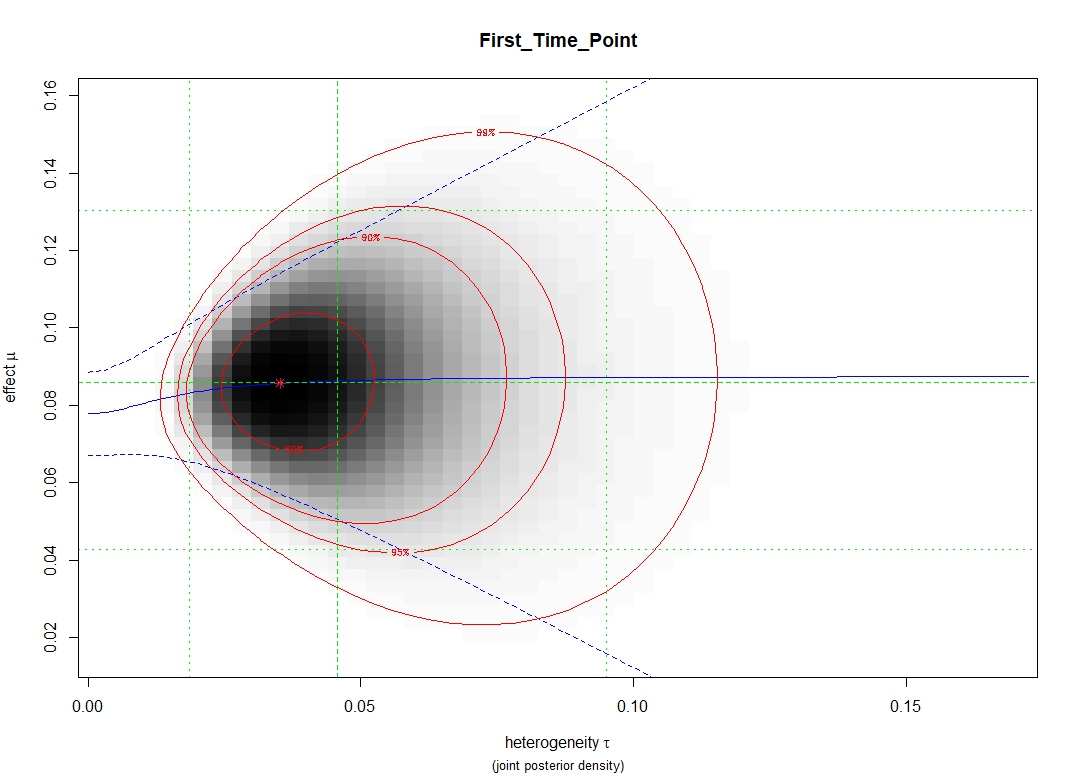


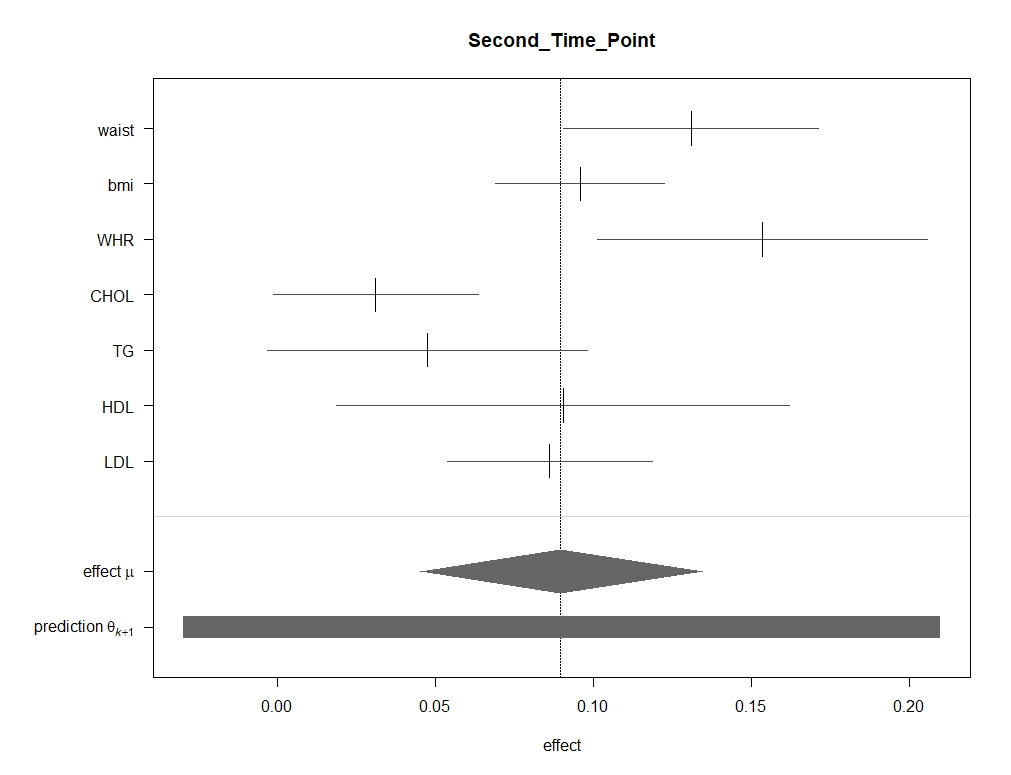

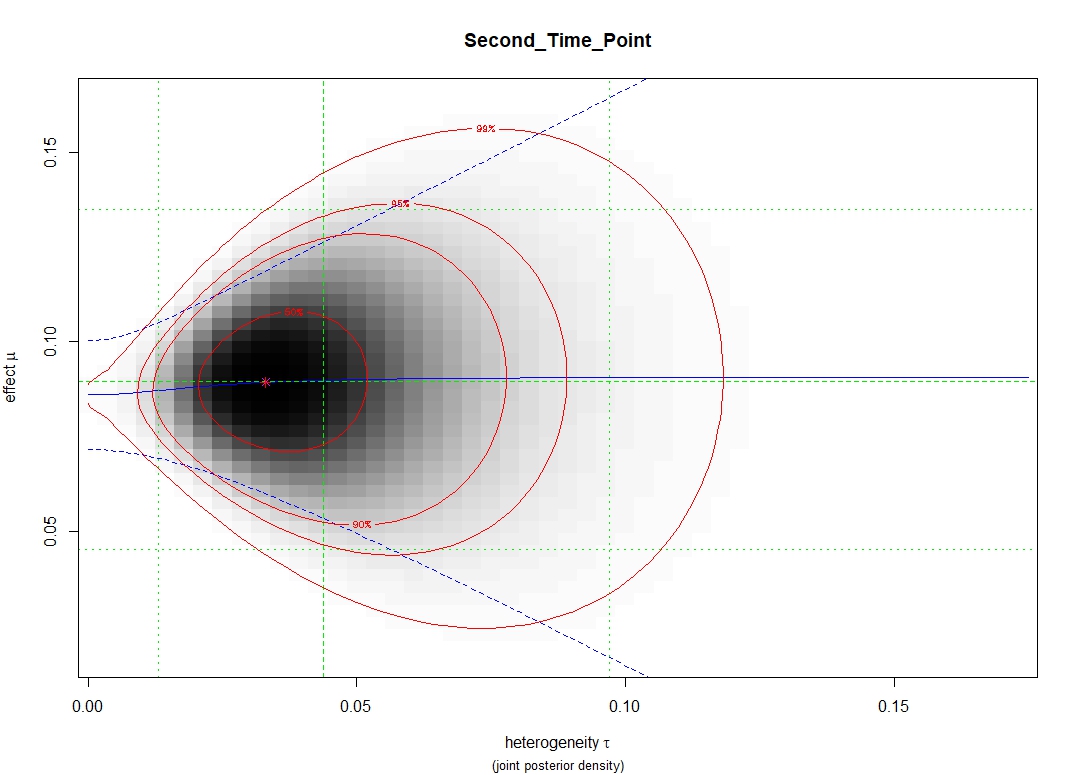


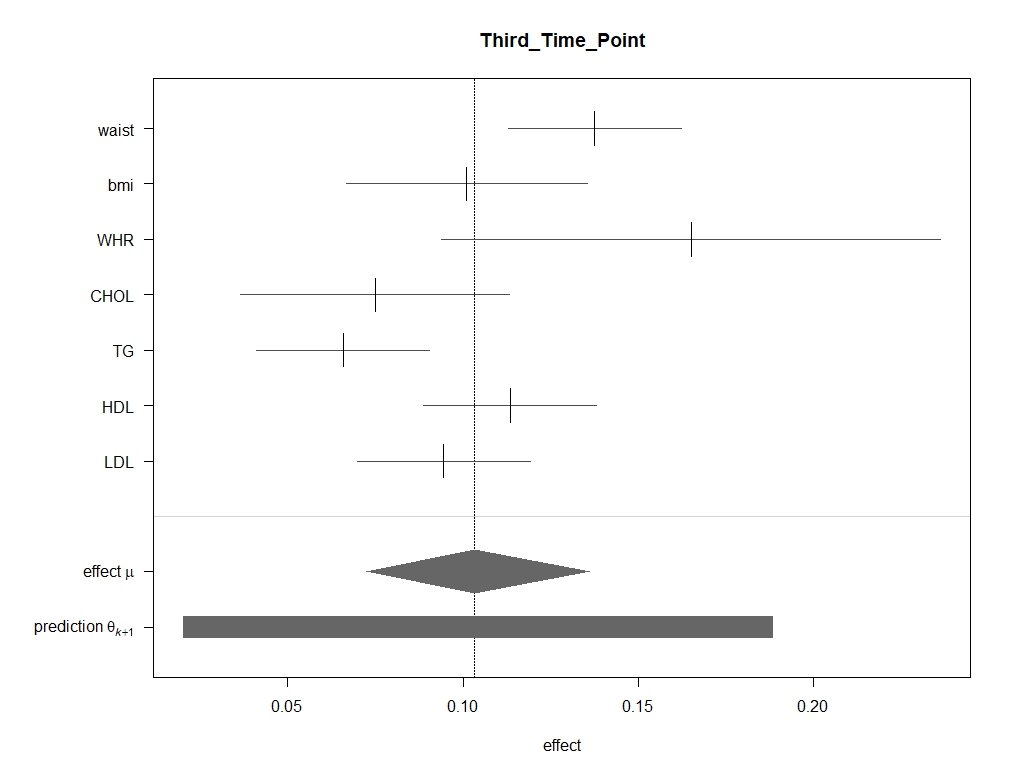


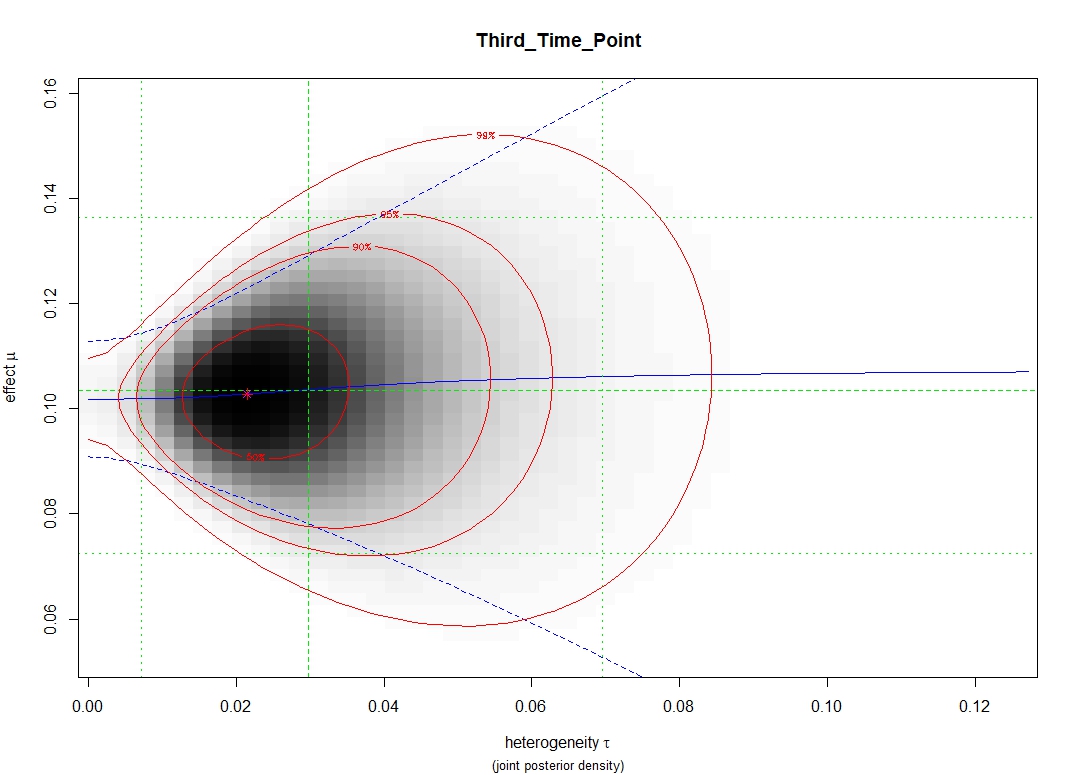


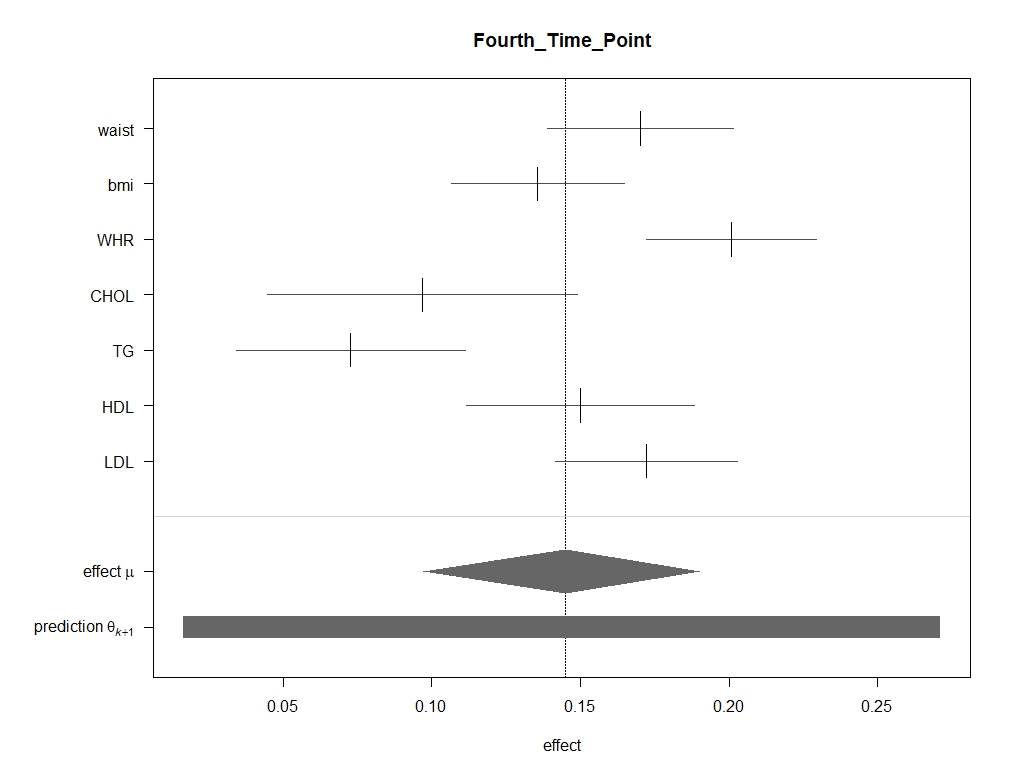


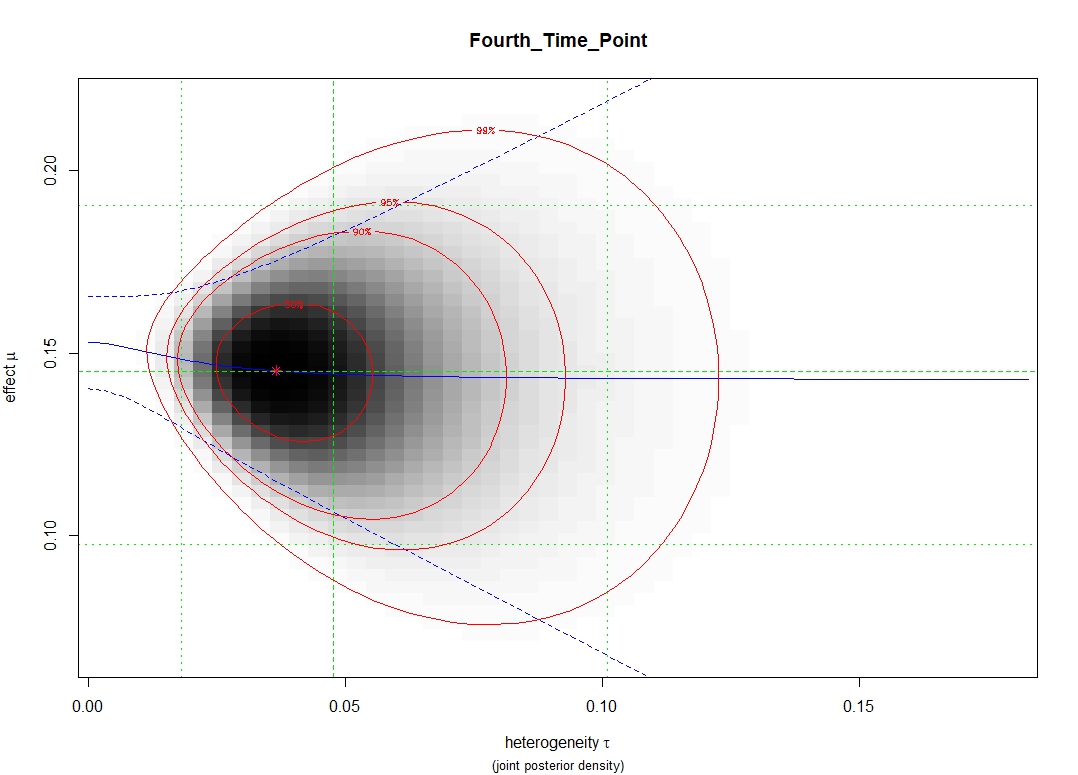


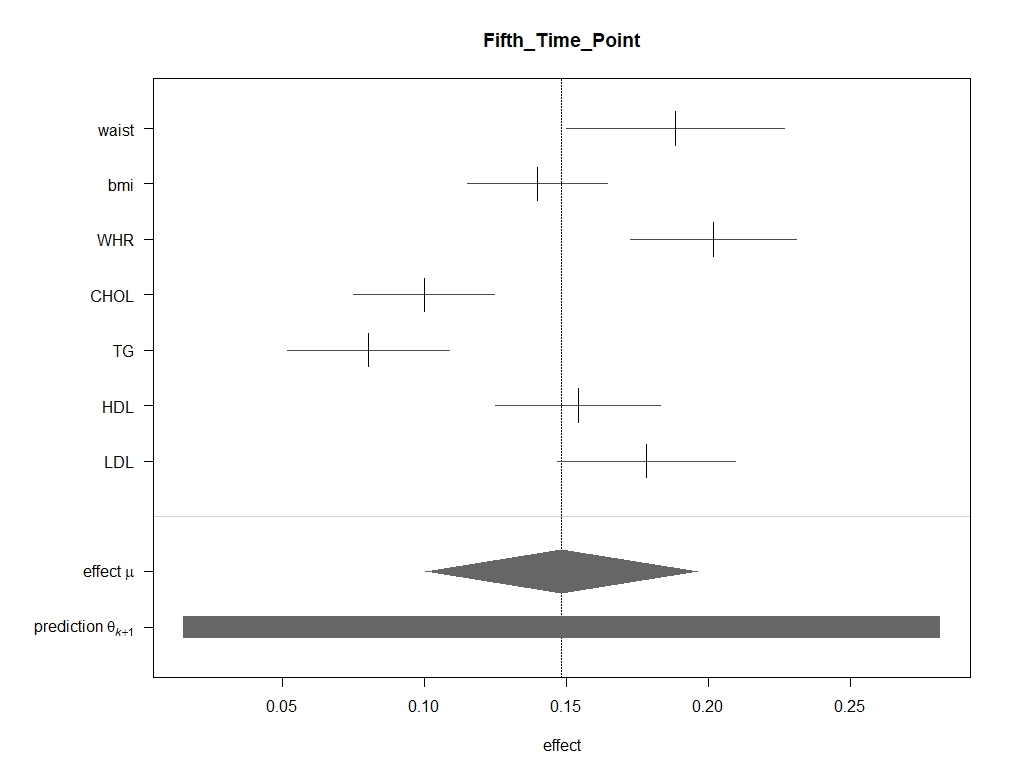


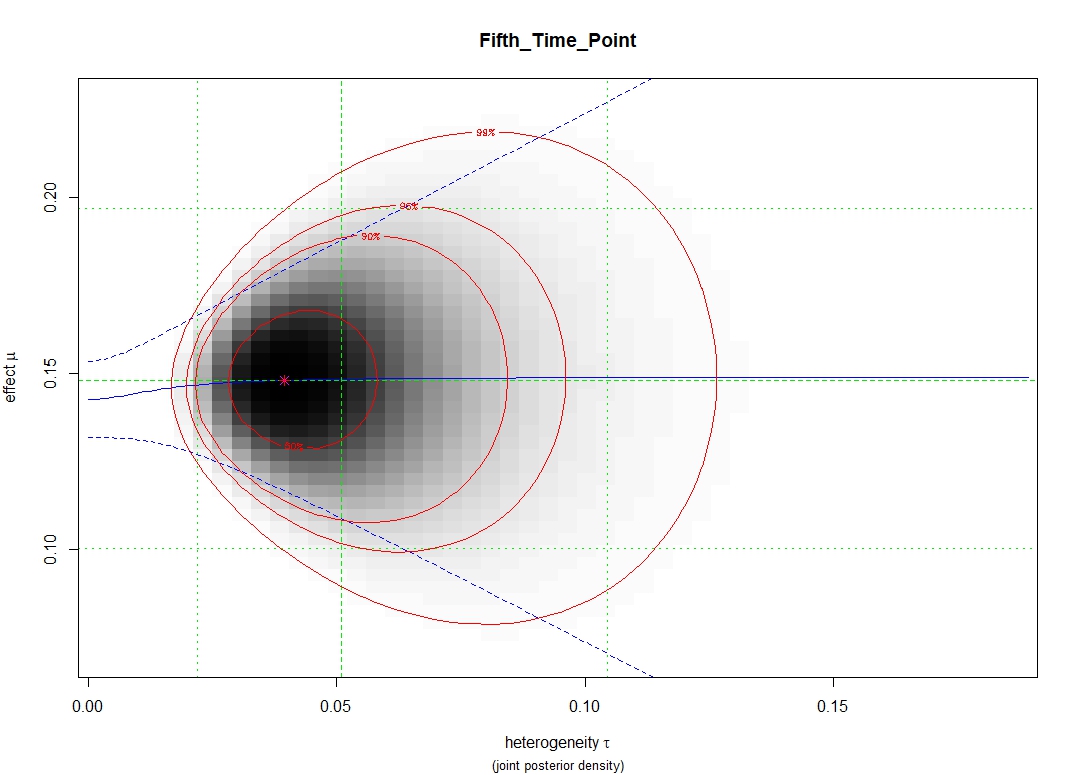

Supplement: Multimedia component 2 [file mmc2.docx]
